# An Efficient Identity-Based Key Management Scheme for Wireless Sensor Networks Using the Bloom Filter

**DOI:** 10.3390/s141017937

**Published:** 2014-10-26

**Authors:** Zhongyuan Qin, Xinshuai Zhang, Kerong Feng, Qunfang Zhang, Jie Huang

**Affiliations:** 1 School of Information Science and Engineering, Southeast University, Nanjing 210096, China; E-Mails: shuaishuaizhang@yahoo.com.cn (X.Z.); fengkerong@163.com (K.F.); jhuang@seu.edu.cn (J.H.); 2 Key Lab of Information Network Security, Ministry of Public Security, Shanghai 201204, China; 3 Computer Department, Nanjing Institute of Artillery Corps, Nanjing 211132, China; E-Mail: zhangqunfang520@163.com

**Keywords:** key management, identity-based cryptography, bloom filter, security, wireless sensor network

## Abstract

With the rapid development and widespread adoption of wireless sensor networks (WSNs), security has become an increasingly prominent problem. How to establish a session key in node communication is a challenging task for WSNs. Considering the limitations in WSNs, such as low computing capacity, small memory, power supply limitations and price, we propose an efficient identity-based key management (IBKM) scheme, which exploits the Bloom filter to authenticate the communication sensor node with storage efficiency. The security analysis shows that IBKM can prevent several attacks effectively with acceptable computation and communication overhead.

## 1. Introduction

Wireless Sensor Networks (WSNs) are ripe for wide adoption in several applications, such as military, healthcare, automotive, research, and so on. For applications such as military, higher requirements on WSN security is needed. However, WSN security is a challenging problem, because of the openness of WSNs' network architectures, which enables adversaries to easily eavesdrop, intercept, inject and alter transmitted information. Besides, the existing computer network security mechanisms cannot be adopted in WSNs because of the restricted node resources and low communication bandwidth. Therefore, it is urgent to put forward low consumption key management schemes for WSNs.

Until now, key management schemes in WSNs were mainly based on symmetric cryptographic algorithms or public key cryptography algorithms. For the symmetric cryptographic algorithm, pool-based key predistribution [1] and the related improved schemes, [2,3] proposed probabilistic key pre-distribution schemes for pairwise key establishment. Their basic idea is that each node randomly picks a set of keys from a key pool before deployment, so that any two sensor nodes have a certain probability to share at least one common key. Blom [4] and Blundo *et al.* [5] proposed a matrix and a polynomial key generation schemes, respectively. However, most of these schemes cost much memory and significant communication overhead with a low security level. On the other hand, the key management schemes based on public key cryptography algorithms could provide much simpler solutions with much stronger security resilience compared with those based on symmetric cryptographic algorithms. However, public key cryptography algorithms require more computing capacity, and for this reason, the public key system was generally considered not applicable for energy-constrained WSNs. Recent works [6–9] have demonstrated the feasibility of public key cryptography algorithms on the resource-constrained sensor nodes. Specially, Oliveira *et al.* [7] implement pairings for sensor nodes based on the 8-bit/7.3828-MHz ATmega128L microcontroller (e.g., MICA 2 and MICAz motes), and they argue that pairing-based cryptography is indeed viable in resource-constrained nodes. Usually, they use Public Key Cryptography (PKC) schemes for bootstrapping security in WSNs, *i.e.*, for generating symmetric keys to communicate or key distribution. TinyPK [6] exploits the RSA cryptosystem to provide authentication and key exchange between an external party and a sensor network, but it needs a certificate authority (CA), and certificates are eliminated instead of the challenge-response protocol in which the public key is signed by CA's private key. Kui *et al.* [10] addressed the multiuser broadcast authentication problem in WSNs by designing PKC-based solutions. Their schemes are built upon the integration of several cryptographic techniques, including the Bloom filter, the Merkle hash tree, *et al.*, However, they use the Bloom filter between the base station and the network user, where the network users refer to personnel or devices that use the WSN; they are not sensor nodes. In our scheme, the Bloom filter is used among the sensor nodes in WSN to provide an efficient authentication.

There are several problems in [6–9]. For example, how does one verify the validness of a public key? Conventional solutions, such as Public Key Infrastructure (PKI) and a certificate, are non-implementable in WSNs, due to their constrained resource. How does one apply Identity-Based Encryption (IBE) in WSNs efficiently and securely with the integrity of a public key? Public key validness is hard to be verified in present IBE schemes, because it usually depends on the certificate and CA. Additionally, the certificate will result in a large communication overhead and expensive signature verification operations, which consume more energy [10].

Because of the absence of PKI and a certificate, there is no authentication in the state-of-the-art IBE schemes, which are subject to many attacks, such as the Sybil attack, the man-in-the-middle attack, *etc.* Focused on addressing these problems, we propose an efficient identity-based key management scheme (IBKM) in this paper, which adopts an identity-based cryptosystem to distribute session keys between nodes without the complicated operations of the public key certificate; specifically, we exploit the Bloom filter to provide authentication with storage efficiency. A Bloom filter is a simple space-efficient randomized data structure based on a hash function for representing a set in order to support membership queries. Although Bloom filters allow false positives, for many applications, e.g., WSNs, the space savings outweigh this drawback when the probability of an error is sufficiently low [11].

The main contributions of this paper are as follows:

(1) To the best of our knowledge, we are the first one to exploit the Bloom filter to authenticate sensor nodes in WSNs. The sensor node's public key in IBE is verified using the Bloom filter together with its ID.
(2) We come up with a security analysis, as well as quantitative memory, computation and communication overhead to demonstrate the effectiveness and efficiency of IBKM. The computation overhead that is brought by the Bloom filter is quite small, as hash operations are negligible compared with the bilinear pairing.

The rest of this paper is organized as follows. Section 2 introduces related works. Section 3 gives some preliminaries on bilinear pairing, bilinear computational Diffie–Hellman (BCDH) and the Bloom filter. We describe IBKM in Section 4. In Section 5, a security analysis of IBKM is given to prove its resilience against various types of attacks. Section 6 gives the evaluation of the storage, computation and communication overhead. Finally, Section 7 concludes the paper.

## 2. Related Works

The notion of identity-based public key schemes was firstly introduced by Adi Shamir [12], who presented an identity-based signature scheme. As compared with the traditional certificate-based public-key cryptosystems, the ID-based system utilizes the users' identity (for example, name or email address) as the public key; therefore, additional computations to verify the corresponding certificates are not needed.

Until now, several cryptography algorithms based on identity have been proposed; however, these solutions cannot completely meet the requirements of practical use, especially in WSNs. In 2001, Franklin and Boneh [13] proposed an identity-based encryption scheme from Weil pairing. They also showed that their scheme can gain security against an adaptive chosen cipher text attack in the random oracle model. Their work is based on bilinear pairings on elliptic curves and led to high research activity in this field.

Yang *et al.* [14] propose an approach based on identity-based encryption and Diffie–Hellman algorithms, which provides authenticated key agreement between pairs of sensor nodes. However, its computation and memory overhead are too high to be practically applied. Zhang *et al.* [15] propose a new security scheme based on LOCK [16] and ID-based secure group key management. They use the exclusion basis system (EBS) [17] for key agreement between the gateway and node, while ID-based key management between the base station and gateway.

Since Boneh *et al.* [18] proposed a signature and encryption scheme based on identity from pairing; many schemes [19–22] attempt to apply pairing on WSNs. Yang *et al.* [22] proposed IBAKA using pairing-based cryptography. Their scheme achieves significant improvements in terms of security strength, communication and storage overhead. Later in this paper, we will compare our scheme with theirs. However, the pairing costs too much computation overhead for WSNs. Barreto [23] proposed an efficient approach to compute pairings on supersingular curves, which can be used for elliptic and hyperelliptic curves with very efficient results. Manel *et al.* [24] propose a scheme based on identity, which supports the establishment of pair-wise keys and cluster keys. However, their scheme does not verify the authenticity of the identity before the key agreement between two nodes; also, nodes in this scheme store a bunch of other nodes' public key and the identity value, which increases the storage overhead. Cheng *et al.* [25] presented EKAES, which is an ID-based key agreement and encryption scheme for WSNs, but their scheme has an expensive communication overhead. Chatterjee *et al.* [26] propose an ID-based key management scheme using bilinear pairings. The nodes in their scheme verify the authenticity of other nodes through the cluster, which causes a heavy communication overhead.

Kui *et al.* [10] presented several public-key-based schemes to achieve immediate broadcast authentication in WSNs, and the Bloom filter is used. However, in their schemes, the broadcast messages are initiated by network users, which are personnel or devices that use the WSN; they are not sensor nodes. Instead, our scheme is used to authenticate among the sensor nodes in a WSN.

Altogether, state-of-the-art identity-based approaches do not verify the authenticity of the corresponding node before the key agreement, because certificate verification usually needs extensive computation, which causes much computation overhead on the sensor nodes; besides, more entities, such as CA, should be setup. In this paper, we address this problem by adopting the Bloom filter with minimized computational and storage costs to cope with the resource-constrained nature of WSNs.

## 3. Preliminaries

In this section, we give a brief introduction to bilinear pairing, the bilinear Diffie–Hellman (BDH) problem and the Bloom filter.

Bilinear Pairing: Let *G*_1_ be a cyclic additive group of prime order *q* and *G*_2_ be a cyclic multiplicative group of the same order *q*. *e*: *G*_1_×*G*_1_→*G*_2_ between these groups is a bilinear pairing if it satisfies the following properties:

1. Bilinear: We say that a map *e*: *G*_1_×*G*_1_→*G*_2_ is bilinear if *e*(*aP*,*bQ*) = *e*(*P,Q*)*^ab^* for all *P*,*Q*∈*G*_1_, while *a*,*b*∈*Z*.
2. Non-degenerate: There exists *P*,*Q*∈*G*_1_, such that e(*P*,*Q*) ≠ 1.
3. Computable: There exists an efficient algorithm to compute e(*P*,*Q*) for all *P*,*Q*∈*G*_1_.

Bilinear Diffie–Hellman (BDH) problem: For given {*P*,*aP*,*bP*,*cP*}, it is a hard problem to compute *e*(*P,P*)*^abc^* for some *a*,*b,c*∈*Z*.

Bloom filter: A Bloom filter [11] is a simple space-efficient randomized data structure; it can be used to succinctly represent a set in order to support membership queries. In our scheme, we use it to authenticate a sensor node while receiving the communication request. A Bloom filter is described by a vector of *m* bits, which are initially all set to zero. In order to represent a set *S* = {*x*_1_,*x*_2_⋯*x_n_*} that contains *n* elements, we use *k* independent hash functions to map each item to the *m*-bits vector. For each element, *x* ∈ *S* bits *h_a_*(*x*) are set to one. Then, we have:

(1) $${\text{BloomFilter}}_{i}=\left\{ \begin{array}{ll} 1, & \text{if}\exists a\in [1,k],b\in [1,n]\\ \;s.t. & \;h_{a}(x_{b})=i\\ 0, & \text{otherwise} \end{array}\right.$$

The initial value: *BloomFilter_i_* = 0 *i* ∈ [1,*m*]

**Figure 1.**
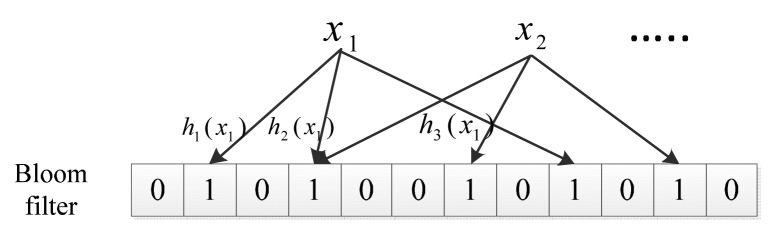
Example of a Bloom filter.

A simple example is shown in Figure 1 when *k* = 3. *x*_1_ is hashed by three hash functions, and three corresponding items in the Bloom filter are set to one. Note that a bit of the vector can be set to one multiple times, but only one works.

## 4. IBKM Scheme

Basically, there are two architectures available for WSNs. One is a distributed flat architecture, and the other is a hierarchical architecture. Considering the limitations of WSNs, such as low energy supply, extremely large network size and redundant low-rate data, the hierarchical network model has more operational advantages than the flat homogeneous model for wireless sensors [27].

**Figure 2.**
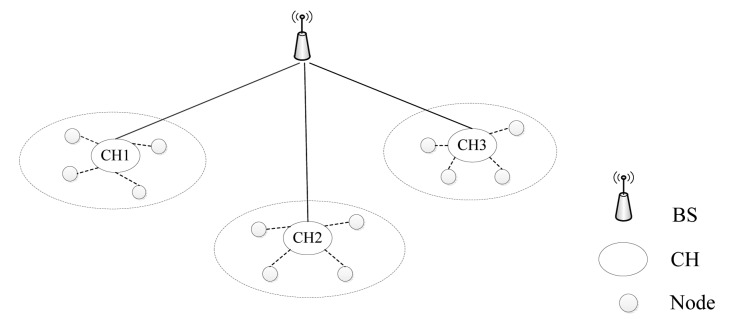
Network model of WSN.

In this work, we focus on the hierarchical network model, which is shown in Figure 2, as in [28]. It has three different kinds of wireless devices; base station (BS), cluster head (CH) and sensor node (N). We assume that the BS is trusted and that the CH is more capable than normal nodes. In a cluster, the CH collects and aggregates packets from its member nodes and then forwards them to the BS. Normally, a member sensor node can transfer packets to CH through several hops.

IBKM consists of three phases: parameters initialization, node registration and share secret key generation between two nodes. Table 1 displays the notations used in this paper.

### 4.1. Parameters Initialization Phase

BS selects large prime *p*, *q* and generates a random elliptic curve E over finite field *F_p_*. One point P on curve E is selected and used as generator to construct an additive group *G*_1_, and $e:G_{1}\times G_{1}\to F_{p}^{*}$ is a bilinear map. H1:{0,1}*→*E*(*F_P_*) are two cryptographic hash functions.

(1) BS selects a random number *s* and computes *P_pub_* = *sP*∈*G*_1_ as the public key. BS broadcasts the public parameters (*G*_1_,*E*(*F_P_*),*p*,*q*,*e*,*P*,*P_pub_*,*H*_1_,*H*_2_).
(2) BS generates each node's ID and calculates the public and private key pair of the node. Then, BS preloads them into the node. The public key is *Q_N_* = *H*_1_(*ID_N_*), and the private key is *S_N_* = *sQ_N_*, where N represents a node in WSNs.
(3) BS generates the CH's ID and calculates the public and private key pair of the CH. Then, BS stores them in the CH, in which the public key is *Q_CH_* = *H*_1_(*ID_CH_*); the private key is *S_CH_* = *sQ_CH_*, where CH is a cluster header in WSNs.
(4) BS keeps a list of all nodes' IDs and their public-private key pairs. BS also keeps all CHs' IDs and public keys for the next steps.

### 4.2. Node Registration Phase

**Figure 3.**
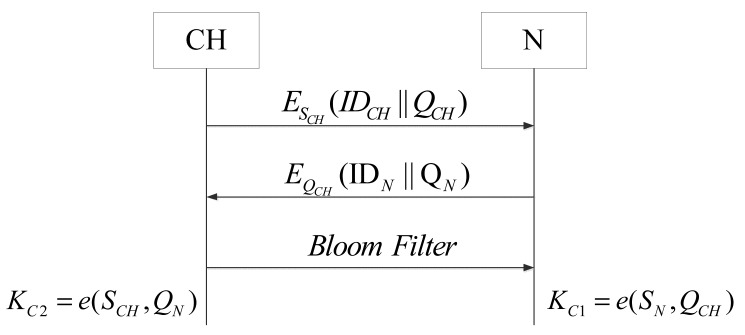
Procedure of node registration.

In this phase, all sensor nodes register to the cluster heads and a session key is generated between each node and their cluster head, as shown in Figure 3.

**Figure 4.**
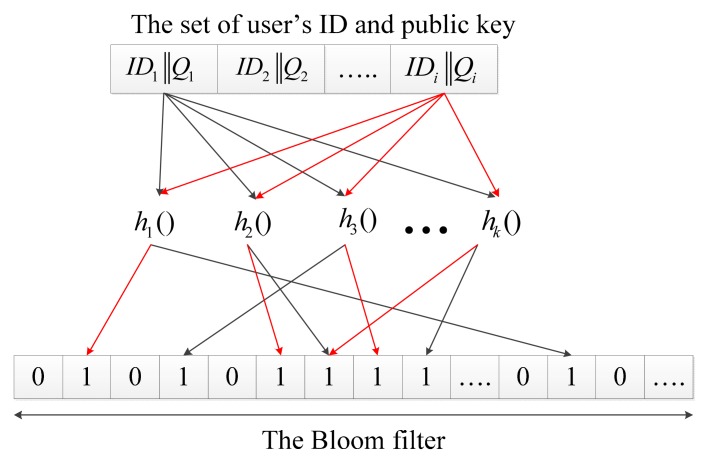
CH generates its own cluster Bloom filter.

(1) CH broadcasts a message that contains its own identity and a public key to all neighboring sensor nodes:
  $$CH\overset{E_{S_{CH}}(ID_{CH}\|Q_{CH})}{\to }\text{All Nodes}$$
(2) Upon the receipt of CH's messages, each sensor node sends its ID and public key to the CH with whom it wants to join.
  $$\text{All Nodes}\overset{E_{Q_{CH}}(ID_{N}\|Q_{N})}{\to }CH$$
(3) After receiving the ID and public key of a node, CH calculates the session key *K_C_*_2_.
  $$K_{C2}=e(S_{CH},Q_{N})$$
(4) Node calculates the same session key with CH.
  $$K_{C1}=e(S_{N},Q_{CH})$$
  *K_C_*_1_ = *K_C_*_2_ can be proved as follows:
  (2) $$\begin{array}{l} \begin{array}{l} K_{C1}=e(S_{N},Q_{CH})=e(S_{N},H_{1}(ID_{CH}))=e(sQ_{N},H_{1}(ID_{CH}))\\ =e(sH_{1}(ID_{N}),H_{1}(ID_{CH}))=e(sH_{1}(ID_{CH}),H_{1}(ID_{N}))\\ =e(sQ_{CH},H_{1}(ID_{N}))=e(S_{CH},H_{1}(ID_{N}))=e(S_{CH},Q_{N})\\ =K_{C2} \end{array} \end{array}$$
  We let *K_C_* = *K_C_*_1_ = *K_C_*_2_
(5) CH generates a Bloom filter of all nodes' IDs and public keys within its cluster and sends the Bloom filter encrypted by the session key generated before to all nodes in the cluster. Figure 4 shows the generation of the Bloom filter.
  $$CH\overset{E_{K_{C}}(\text{Bloom Filter})}{\to }\text{All Nodes}$$

### 4.3. Share Secret Key Generation between Two Nodes

**Figure 5.**
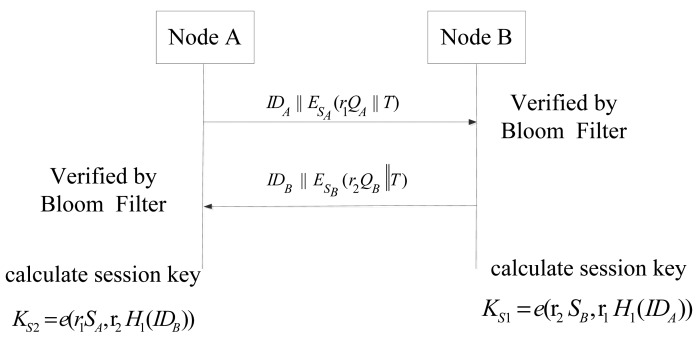
Share secret key generation between two nodes.

(1) Sensor Node A chooses a random number *r*_1_ and broadcasts a message that contains its ID, public key and a time stamp encrypted by its own private key to neighboring nodes after it registers to the CH.
  $$A\overset{ID_{A}\|E_{S_{A}}(r_{1}Q_{A}\|T)}{\to }\text{Neighbor Nodes}$$
(2) When the neighboring Node B receives the message, it verifies the authenticity of A by checking if the hash mapping of (*ID_A_*, *Q_A_*) is contained in the Bloom filter obtained from CH. A negative answer means authentication failure. Our node authentication algorithm takes a similar idea as in [10] and is provably efficient. If the authentication is passed, B chooses its random number *r_2_* and returns its ID, public key and a time stamp encrypted by its own private key. Then, B calculates the session key *K_S_*_1_.
  $$\begin{array}{c} B\overset{ID_{B}\|E_{S_{B}}(r_{2}Q_{B}\|T)}{\to }A\\ K_{S1}=e({\text{r}}_{2}S_{B},{\text{r}}_{1}\;H_{1}(ID_{A})) \end{array}$$
(3) A decrypts the message and get B's ID and public key with its own private key and verifies the authenticity of B using the Bloom filter obtained from CH. If B is authenticated, A calculates the session key *K_S_*_2_.
  Thanks to the properties of the bilinear map, we can prove *K_S_*_1_= *K_S_*_2_ as follows.
  (3) $$\begin{array}{l} K_{S1}=e({\text{r}}_{2}S_{B},r_{1}H_{1}(ID_{A}))=e({\text{r}}_{2}sQ_{B},r_{1}H_{1}(ID_{A}))\\ =e({\text{r}}_{2}sH_{1}(ID_{B}),r_{1}H_{1}(ID_{A}))=e(r_{1}sH_{1}(ID_{A}),{\text{r}}_{2}H_{1}(ID_{B}))\\ =e(r_{1}sQ_{A},{\text{r}}_{2}H_{1}(ID_{B}))=e(r_{1}S_{A},{\text{r}}_{2}H_{1}(ID_{B}))=K_{S2} \end{array}$$
  Afterwards, Nodes A and B can communicate with each other using the shared session key. The shared secret key between two nodes can be decided as shown in Figure 5.

## 5. Security Analysis

Due to the unreliable wireless channel and volatile topology, a key agreement scheme for WSNs is subject to various attacks, such as node-compromise attack, Sybil attack, *etc.* Compared to previous works, our scheme can resist these attacks using the bilinear map and authentication through the Bloom filter.

Sybil Attack: Before node deployment, the BS allocates an ID for each node in the WSNs, and then, the CH generates a Bloom filter of nodes in its own cluster. Therefore, before sharing the secret key between two nodes, they authenticate each other using the Bloom filter generated by CH. Therefore, IBKM can resist Sybil attack because an adversary cannot convince another node that it has a legal ID.

Node-compromise attack: It is easy to capture a node in WSNs and steal secret information about the network stored in the node. Compared to the E-G and other key pre-distribution schemes, IBKM can resist node-compromise attack and ensure the security of the entire network. For the E-G scheme and its variants, if the number of node adversaries captured exceeds a certain threshold, the adversaries will get almost all of the keys of the WSN. However, in our scheme, different node pairs share different keys; even if a node is compromised, it will not affect other node pairs' keys.

Rekeying and forward secrecy*:* IBKM employs a random number r in the process of secret key generation between two nodes. On the one hand, we can stipulate the secret key agreement period; therefore, nodes must renegotiate a new session key in a certain period. In this way, we can enhance the security of the network. On the other hand, the rekeying can provide forward secrecy of the network when a node is captured by the adversary. Even if the adversary gets the current secret key, he cannot deduce the keys used before, because different random numbers generate different secret keys.

HELLO flood attack: In this attack, the main aim of the attacker is to deplete the node energy. In our scheme, every node possesses a Bloom filter for node identity authentication. Therefore, if an adversary sends a HELLO message, the receiver nodes will first check if the message is legitimate or not. If the result is negative, later calculation will not be carried on. Therefore, no more energy of the received node will be consumed.

Man-in-the-middle attack: In our scheme, the adversary cannot calculate the pairwise session key, even if it intercepts the system parameters, since the messages transmitted in our scheme are all encrypted in the public key cryptosystem. On the other hand, the session key is generated by the private key and the random number. It is assumed to be hard for an adversary to decrypt the message on air or to calculate the session key.

Mutual authentication: Our scheme achieves both identity authentication and key authentication. Before the session key is agreed upon, the nodes verify the authenticity of each other by checking if the corresponding hash mapping is contained in the local Bloom filter. A negative answer means that the node is illegal in this cluster. Then, we verify the identity of the node by the signature of the private key. While, after, Node A and Node B share the same session key, they can realize identity authentication by the session key, because only A and B share the same key. In this way, we can prevent the unauthenticated node from accessing the sensor network.

## 6. Performance Evaluation

Although security is a critical factor in WSNs, it is also necessary to evaluate the storage, computation and communication consumption of sensor nodes, since they are extremely resource constrained. In this section, we evaluate the performance of IBKM by comparing the storage, computation and communication overhead with two relevant schemes, Yang's scheme [22] and Cheng's scheme [25]. The two schemes were influential ones of the key management protocols proposed for WSNs.

### 6.1. Performance of Bloom Filter

Since we apply the Bloom filter to provide probabilistic membership verification in our scheme and hash functions have the disadvantage to collision, it is important to evaluate the probability of the false positive.

Theorem 1: Given the number of cluster nodes *n* and the storage space *m* bits for a single Bloom filter, assume the number of hash functions in the Bloom filter is *k*; we can get the minimum probability of the false positive *f* = 2^−^*^k^* with the number of hash functions around $k=(\overset{m}{\;}/\underset{n}{\;})\ln2$.

Proof: Since *f*=(1−(1−1/m)*^kn^*)*^k^* ≈ (1−*e*^−^^*kn*/*m*^) [10], we can have *f* = *e*^*k* ln(1−e^−*kn*/*m*^)^. Let *g* = *k* ln(1−e^−*kn*/*m*^); hence, minimizing *f* is equivalent to minimizing *g* with respect to *k*. We find:

(4) $$\frac{dg}{dk}=\ln(1-e^{-kn/m})+\frac{kn}{m}\frac{e^{-kn/m}}{1-e^{-kn/m}}$$

It is easy to check that the derivative is zero when $k=(\overset{m}{\;}/\underset{n}{\;})\ln2$. We substitute $k=(\overset{m}{\;}/\underset{n}{\;})\ln2$ into *f* = (1−*e*^−^*^kn^*^/^*^m^*)*^k^* and get *f* = 2^−^*^k^*.

**Figure 6.**
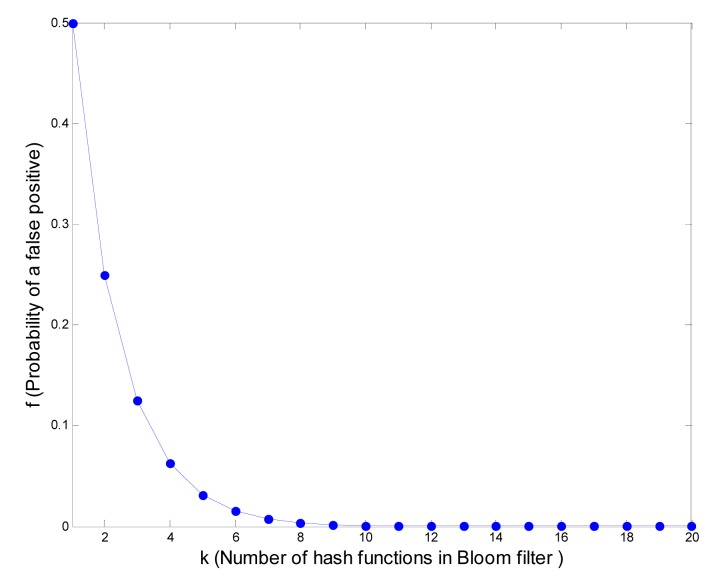
Minimum probability of false positives.

In addition, it is not hard to show that this is a global minimum [11]. Now, we can see that the probability of a false positive *f* is a function of *k*, *i.e.*, *f* = 2^−^*^k^*. Figure 6 shows that as *k* increases, the false positive decrements rapidly. Then, we can choose appropriate *k* according to the different application scenarios of WSNs to achieve an acceptable false positive rate. In our following experiments, we take *k* as 10, since the false positive *f* has dropped below 10^−3^.

### 6.2. Memory Overhead

**Figure 7.**
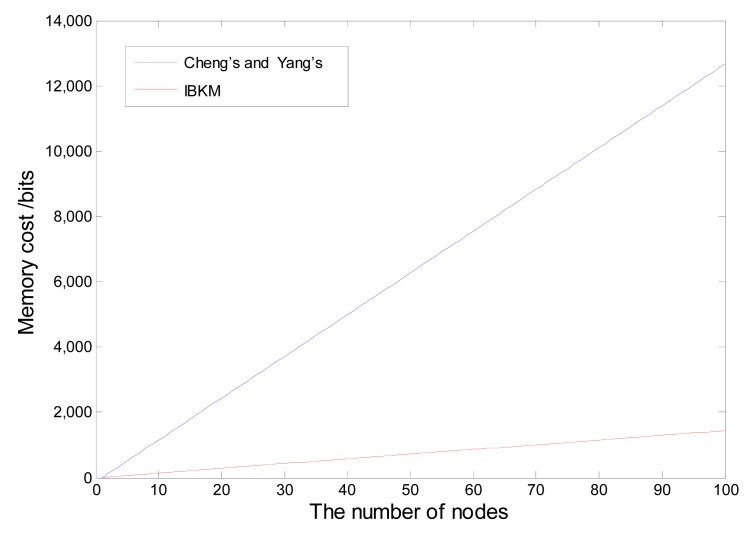
Comparison of memory overhead. IBKM, identity-based key management.

It can be obtained from the above analysis that the probability of the false positives reaches the minimum when *m* = *n*(*k*/ln 2), and the minimum value is *f* = 2^−^*^k^*. For a certain network whose number of nodes *n* is determinate, thus the probability of the false positive changes after *m* and *k*. To maintain the minimum probability of the false positives, we should keep *m* = *n*(*k*/ln 2). It turns out that when we want smaller *f*, we should use larger *m* or *k*, which means more memory consumption and more hash computation; therefore, we should make a trade-off to choose appropriate *k* and *m* in accordance with different scenarios. At this point, we assume a civilian scenario in which *f* is acceptable when less than 1%, *i.e.*, *k = 10*, we obtain m=14.427*n. In Cheng's and Yang's scheme, each node is preloaded with other node's public keys, while our scheme only use the Bloom filter to verify the public keys. Therefore, for convenience, to compare the three schemes, we take the whole cluster memory consumption as the measurement. Here, we assume the public key is 128 bits long; thus, the total memory of preloaded keys in the cluster is m=128*n for Cheng's and Yang's scheme. Figure 7 shows the performance comparison of IBKM, Cheng's and Yang's schemes. From the comparison, we can see that our scheme costs significantly less memory consumption than their schemes.

### 6.3. Computational Overhead

We implement our proposed scheme in Microsoft Visual C++ 6.0. The operating system is Windows 7 Ultimate. The computer configuration is as follows: CPU, Intel Core i5 3.2 GHz; memory, 4 GB; hard disc, 1 TB. In our scheme, we need to compute one bilinear pairing, exponentiation on *G_T_*, 160-bit scalar point multiplication and the Bloom filter. Yang's scheme [22] involves the computation of two bilinear pairings, one exponentiation on *G_T_* and one 271-bit scalar point multiplication. Cheng's scheme [25] involves the computations of two bilinear pairings, one exponentiation on *G_T_*, two 160-bit scalar point multiplications and one 271-bit scalar point multiplication. We calculate the time needed for the major operations, which is shown in Table 2. From this, we can see that the most time-consuming operations are bilinear pairing, exponentiation on *G_T_* and 160-bit point multiplication. The Bloom filter only costs about 1/86, 1/9 and 1/6 of the bilinear pairing, exponentiation on *G_T_* and point multiplication, respectively.

A comparison of total computation overhead with the two schemes is shown in Table 3. From the table, we can see that, since we only use one bilinear pairing, which is the most time-consuming operation, our scheme needs less computation time than the other two schemes; therefore, IBKM saves the energy consumption of node. Notice that, although hash mapping and encryption are introduced in the Bloom filter, they are omitted, since they consume negligible computing power compared with that of bilinear pairing, *etc.*

### 6.4. Communication Overhead

In IBKM, the secret key generation process comprises two messages: one is sent by A, and the other is sent by B. Each message includes the node's ID, public key and a time stamp, namely the message is *ID*∥*E*(*rQ*∥*T*). In this message, rQ is a point on elliptic curve, which given x of rQ, the node can derive y whenever it needs. In accordance with Yang's scheme, Q can be compressed to 34 bytes and two bytes for ID. We take eight bytes for the time stamp in our scheme, and the encryption here does not change the length of the messages. Therefore, the communication overhead of our scheme is 44 bytes. While Yang's scheme needs 61 bytes for the message of 〈*U*,*V*〉 and Cheng's scheme needs 168 bytes for using the 1024-bit modular in Diffie–Hellman key exchange, their schemes both need two messages when nodes share a secret key in the communication process. We show the communication overhead of the three schemes in Table 4. From this table, we can see the superiority of our communication consumption compared with the other two schemes.

## 7. Conclusions

In this paper, we propose IBKM, which is an efficient key management scheme for WSNs. By adopting the Bloom filter into identity-based cryptosystem to distribute session keys between nodes, IBKM achieves the advantages of the node's identity authentication without complex certification verification by the certification authority. The results of the analysis show that our scheme can resist various attacks and has acceptable overhead in storage, computation and communication compared to the existing related schemes.

## Figures and Tables

**Table 1.** List of the notations used.

**Notation**	**Description**
*_G_*_1_	A cyclic additive group of prime order *q*
*_G_*_2_	A cyclic multiplicative group of prime order *q*
*E*	A random elliptic curve
*P*	A point on *E*
*p*, *q*	Large prime numbers
*e*( )	A bilinear mapping function
*H*( )	Hash function
*E_k_*( )	Symmetric encryption with key *k*
T	A time stamp
*r*	Random number
*ID_CH_*	Identity of cluster head *CH*
*ID_A_*, *ID_B_*	Identity of node *A* and node *B*
*Q_CH_*, *S_CH_*	Public key and private key of *CH*
*Q_A_*,*S_A_*	Public key and private key of Node *A*
*K_C_*_1_,*K_C_*_2_	Shared secret key between cluster head and node
*K_S_*_1_,*K_S_*_2_	Shared secret key between two nodes in a cluster

**Table 2.** Time consumption of major operations.

**Operations**	**Time/ms**
Bilinear pairing	14.193
Exponentiation on *G_T_*	1.525
point multiplication	0.940
Bloom filter	0.165

**Table 3.** The comparison of computation overhead in the secret key generation of two nodes.

**Schemes**	**Bilinear Airing**	**Exponentiation on*G_T_***	**Point Multiplication**	**Time/ms**
Yang's	2	1	1	30.951
Cheng's	2	1	3	32.831
IBKM	1	0	2	16.438

**Table 4.** The comparison of communication overhead in the secret key generation of two nodes.

**Scheme**	**Cheng's**	**Yang's**	**IBKM**
Communication overhead (bytes)	336	122	88
